# Effects of Male Defendants’ Attractiveness and Trustworthiness on Simulated Judicial Decisions in Two Different Swindles

**DOI:** 10.3389/fpsyg.2019.02160

**Published:** 2019-09-26

**Authors:** Qun Yang, Bing Zhu, Qian Zhang, Yuchao Wang, Ruiheng Hu, Shengmin Liu, Delin Sun

**Affiliations:** ^1^Institute of Psychological Sciences, Hangzhou Normal University, Hangzhou, China; ^2^Mental Health Center, Hangzhou Dianzi University, Hangzhou, China; ^3^Department of Psychology, Huzhou Normal University, Huzhou, China; ^4^Duke-University of North Carolina at Chapel Hill Brain Imaging and Analysis Center, Duke University, Durham, NC, United States

**Keywords:** facial attractiveness, facial trustworthiness, defendants, swindles, judicial decision making

## Abstract

The present study aimed to examine the effects of male defendants’ facial appearance (attractiveness and trustworthiness) on judicial decisions in two different swindles. We selected the following four categories of faces by manipulating facial attractiveness and trustworthiness simultaneously: the attractive and trustworthy face; the attractive but untrustworthy face; the unattractive but trustworthy face; and the unattractive and untrustworthy face. A total of six hundred and sixty-three participants across two studies were asked to make conviction-related judgments and penalty-related decisions for the defendants after they were randomly assigned to one of the four categories of faces. In Experiment 1, we used a blind-date swindle and found a “beauty penalty” for physically attractive defendants among females. Specifically, female participants were more likely to issue a guilty verdict to better-looking male defendants. Additionally, this “beauty-penalty effect” was merely observed in the untrustworthy condition. In Experiment 2, we used a telecommunication swindle, and the results showed that facial trustworthiness significantly predicted punishment magnitude and sentence decisions. Moreover, an exploratory analysis revealed that the disgust evoked by the faces partially mediated the relationship between facial trustworthiness and the assignment of criminal penalties. Taken together, these findings indicated that facial attractiveness and trustworthiness played different roles in judicial decisions. Importantly, the effect of facial attractiveness on judicial decisions differed as the detailed criminal circumstances of the offenses changed.

## Introduction

Judicial decisions at a trial concern individual rights and sometimes even life and death. Therefore, such decisions are expected to be based on deliberative and logic reasoning. However, neither the court judges nor the juries are perfectly rational decision-makers. It has been demonstrated that decision making in legal contexts is not purely logical and instead can be affected by various extralegal factors (Vidmar, 2011; Peer and Gamliel, 2013). The physical appearance of a suspect, such as facial attractiveness and trustworthiness, is one of the most salient heuristic factors that might bias legal decision making (Ahola et al., 2010; Funk and Todorov, 2013).

Facial attractiveness usually increases one’s pleasantness and prompts others to approach him or her favorably. Individuals with attractive faces are believed to have more positive personality traits, a commonly known stereotype called “beauty-is-good,” which can exert significant influences on a variety of aspects in social lives (Eagly et al., 1991). For example, better-looking employees were more likely to get promoted in the company (Marlowe et al., 1996); attractive players received more money from their partners than unattractive responders in an economic decision game (Maestripieri et al., 2017); and attractive candidates were perceived as more politically competent than unattractive ones and were more likely to gain support from the voters in an election (Milazzo and Mattes, 2016; Palmer and Peterson, 2016). However, in legal cases, it remains controversial whether it is advantageous for defendants to have attractive faces.

In the early laboratory work on mock jurors in criminal cases, researchers found an “attraction-leniency effect” on legal decision making by showing that attractive criminal suspects were less likely to be convicted and were given less severe punishments than their unattractive counterparts (Efran, 1974; Leventhal and Krate, 1977). This “attraction-leniency effect” was later observed in the actual court trials in which physical attractiveness ratings showed significant negative correlations with the severity of the sentences imposed by professional judges (Stewart, 1980, 1985). However, the effect of facial attractiveness on legal judgments can be tempered by the nature of the crimes (Sigall and Ostrove, 1975; Smith and Hed, 1979; Goodman-Delahunty and Sporer, 2010). According to Sigall and Ostrove (1975), the leniency effect was only expected in crimes (for example, burglary) that were unrelated to facial attractiveness. It could be replaced by a “beauty-penalty effect” if the crimes were attractiveness-related, such as in the crime of swindling. Specifically, attractive defendants were considered more responsible for their actions or should deserve harsher penalties because they were believed to be able to take advantage of their physical appearance to perform the swindling (Sigall and Ostrove, 1975; Wuensch et al., 1993; Shechory-Bitton and Zvi, 2014). However, the research findings concerning the effect of physical attractiveness on the judicial decisions in swindling are not always consistent. For instance, some showed that attractive suspects were punished less severely in committing a swindle (Wuensch et al., 1991). Others did not find any effect of facial attractiveness on punishments (Smith and Hed, 1979; Mazzella and Feingold, 1994; Shechory-Bitton and Zvi, 2014). Therefore, it is not yet clear how facial attractiveness affects legal judgments in swindling cases.

Facial trustworthiness is another common quality of physical appearance that might be easily taken as a source of information in social interactions. For example, adult participants were inclined to place more trust in partners who appeared to be trustworthy than in those who did not appear to be trustworthy when deciding which person to invest in (van ’t Wout and Sanfey, 2008; Duarte et al., 2012; Rezlescu et al., 2012). Even 10 years old children were affected by facial trustworthiness when making investment decisions (Ewing et al., 2015). Additionally, facial trustworthiness has been shown to be judicially relevant. Mock jurors needed significantly less evidence to reach a guilty verdict when the face of a defendant appeared to be more untrustworthy in severe crimes, such as in murder cases (Porter et al., 2010). Even during judicial activities in the court, perceptions of facial trustworthiness could be used to predict decisions about whether to sentence a convicted murderer to death or life imprisonment (Wilson and Rule, 2015). Surprisingly, hypothetical sentences assigned to the actual murderers solely based on inferences of facial trustworthiness by naive participants predicted the actual court sentences imposed on these capital inmates by well-informed juries. According to the researchers, the visual appearance of trustworthiness, rather than other facial characteristics (i.e., Afrocentricity, attractiveness, and maturity), accounted for the results (Wilson and Rule, 2016). The reason for the biasing effect of facial trustworthiness on judicial decisions might be due to the activation of criminal stereotypes. Trustworthiness and criminal appearance were closely related. Untrustworthy faces were generally perceived as more criminal in appearance than trustworthy faces; faces rated high in criminal appearance were perceived as less trustworthy (Flowe, 2012).

It is noteworthy that facial attractiveness and trustworthiness are highly interrelated with each other. Individuals across a wide range of age groups used certain common facial clues, such as the chin, nose, and brow bridge, in judging whether a face was attractive and trustworthy. Facial attractiveness perceptions were positively related with facial trustworthiness evaluations and could explain a substantial amount of variance in judgments of trustworthiness (Xu et al., 2012; Ma et al., 2015, 2016). As a result, it is of significance to take into account both of them when studying how facial attractiveness or trustworthiness may guide social decision making.

In the present study, the purpose is mainly twofold. First, we found that some studies combined facial attractiveness and trustworthiness and compared the effect of the attractive and trustworthy faces to that of the unattractive and untrustworthy faces on social decision making (Putz et al., 2016, 2018), while other studies focused on the influence of one facial dimension on judicial decisions by including other facial dimensions as covariates (Porter et al., 2010). However, we are particularly interested in examining the interactive effect of facial attractiveness and trustworthiness on judicial decisions and thus manipulating both of the facial features of hypothetical male defendants in our study.

Second, previous findings have been contradictory with regard to the “attraction-leniency effect” or “beauty-penalty effect” of facial attractiveness during judicial judgments in swindling cases. Little research thus far has offered a sound explanation for the inconsistent results across studies. We assumed that this facial attraction effect on judicial judgments might depend on the nature of different swindling crimes. We used two different but comparable swindles – namely a blind-date swindle case in Experiment 1 and a telecommunication swindle case in Experiment 2 – in an attempt to test whether the “beauty penalty” would only occur in the case in which the suspects are able to take advantage their appealing looks to commit the crime of swindling.

Since there has been some evidence on gender differences with regard to the facial effect on judicial decision making (Castellow et al., 1988; Efran, 1974; Wuensch et al., 1991), we recruited a similar number of male and female participants in case a gender difference might exist.

## Experiment 1

In Experiment 1, we examined the effect of physical attractiveness and trustworthiness on the judicial decisions of mock jurors in a blind-date swindle. We adapted the criminal scenario from a real case in which the suspect was accused of swindling a woman out of 10,000 ¥ (approximately $ 1410) by pretending to develop a romantic relationship with the woman. We conjectured that it should be beneficial to be physically attractive to commit fraudulent activities in a romance scam. Thus, judicial decision makers might automatically associate attractive suspects with the strong possibility of taking advantage of their physical appearance to cheat others. Therefore, defendants with attractive faces might suffer a “beauty penalty.” In other words, they might be more likely to receive a guilty verdict and harsher penalties as a result of their better-looking appearances. Moreover, we expect this “beauty penalty” to occur merely when the defendants seemed untrustworthy because a trustworthy face was likely to be associated with less severe penalties and might counteract the negative effect of physical attractiveness on conviction and punishment judgments in this case (Wilson and Rule, 2015, 2016).

### Materials and Methods

#### Participants

A total of 333 undergraduate and graduate students from Hangzhou Normal University voluntarily participated in the experiment. All participants were Asian Chinese, and they were instructed to use their cellphone to take part in an online survey^1^ in a public elective course. Twenty-two participants did not finish all questions and were removed from further data analysis, leaving a final sample of 311 (172 females and 139 males). The age of the sample was between 18 and 29 years old (*M* = 20; *SD* = 1.99). As we focused on the legal decision making of laypersons, students from psychology or law majors were excluded from the present study. We received oral informed consent from all participants prior to participation. The experimental protocol was approved by the Ethics Committee of Hangzhou Normal University and in accordance with the Declaration of Helsinki.

#### Materials

##### *Photograph*s

We initially selected 16 photographs from the internet of Asian Chinese males with neutral expressions, which included a wide range of attractiveness and trustworthiness. All the photos were unfamiliar to the experimenters and the participants. They were further processed to be gray images with only the head and shoulder reserved and were adjusted to be uniform in size [(188–192) ^∗^ (250–257) pixels (220 pixels/inch)].

We conducted a pilot study before the formal experiment to rate the facial attractiveness and trustworthiness of the male faces in the photos. Given that the two ratings might influence each other, two independent samples of participants from the same ethnic group as the defendants portrayed in the photographs (N_1_ = 37, *M*_age_ = 23, *SD* = 0.88; N_2_ = 36, *M*_age_ = 23, *SD* = 1.27) were recruited to rate the attractiveness and trustworthiness of each face on a 7-point scale (1= absolutely not attractive or trustworthy, 7 = extremely attractive or trustworthy). All 16 photos were presented in random order.

Participants were instructed to read the definition of the two facial dimensions before providing their ratings. “Attractiveness” indicates the extent to which a face is beautiful and pleasant. “Trustworthiness” indicates the extent to which an individual could be trusted based upon facial appearance. Participants were asked to make the judgments intuitively. Then, the mean rating scores of each face, as well as the whole set of 16 faces for the two facial dimensions, were calculated. A photo was categorized as an attractive or trustworthy face if its rating scores were higher than the total average score of the 16 photos (*M*_A_ = 3.81, *SD* = 1.23; *M*_T_ = 4.18, *SD* = 0.83), while it was considered an unattractive or untrustworthy face if its rating scores were lower than the total average score.

Finally, four photos were selected as our formal experimental stimuli: an attractive and trustworthy face (*M*_*A*_ = 4.43, *SD* = 1.17; *M*_*T*_ = 4.83, *SD* = 1.21); an unattractive but trustworthy face (*M*_*A*_ = 2.73, *SD* = 1.00; *M*_*T*_ = 4.22, *SD* = 1.27); an attractive but untrustworthy face (*M*_*A*_ = 4.11, *SD* = 1.52; *M*_*T*_ = 3.78, *SD* = 1.29); and an unattractive and untrustworthy face (*M*_*A*_ = 2.16, *SD* = 1.10; *M*_*T*_ = 2.72, *SD* = 1.26).

##### *Criminal cas*e

The criminal scenario used in the formal experiment described a case of a swindle that was adapted from a real case (the detailed criminal case can be seen in the Supplementary Materials). The description of the whole case included the victim’s statements, a preliminary police investigation, and the basic information of the suspect (gender, age, nationality, educational background).

#### Design and Procedure

The present study adopted a 2 (attractiveness: attractive, unattractive) ^∗^ 2 (trustworthiness: trustworthy, untrustworthy) ^∗^ 2 (gender: female, male) between-subjects design. Considering that participants might consciously associate facial appearances with their decision making or identify our experimental goals when seeing photos with different levels of facial attractiveness and trustworthiness, each participant was randomly assigned to only one of the four photos. Eventually, there were 78 participants (44 females) in the attractive and trustworthy face condition, 77 (43 females) in the unattractive but trustworthy face condition, 77 (41 females) in the attractive but untrustworthy face condition and 79 (44 females) in the unattractive and untrustworthy face condition.

Participants were first instructed to envisage themselves as a judge. After reading the descriptions of the case, they were then asked to indicate their dichotomous verdict (guilty or not guilty) and their confidence in that verdict on a 7-point scale (1 = not confident at all, 7 = extremely confident) (see Supplementary Materials for confidence-related findings). Once participants made a guilty verdict, they were further asked to decide on the magnitude of punishment (ranging from 1 to 9, with 1 indicating no punishment and 9 indicating extremely severe punishment).

According to previous literature, people tend to be emotionally positive and willing to approach when faced with attractive faces (Rhodes, 2006; Wang et al., 2015). In other words, highly attractive faces often accompany pleasant emotions. Therefore, to ensure the validity of our manipulation for facial attractiveness, participants were asked to evaluate how pleasant they felt when seeing the photograph following the legal judgments (postexperimental evaluations). Subsequently, participants were asked to complete the criminal appearance scale, a 7-point scale which was used for participants to rate the degree to which a person looked like an offender. The whole scale was composed of 5 items (how much a person looked like a criminal or appeared to be dangerous, aggressive, trustworthy, and honest) (Funk and Todorov, 2013). The scale was found to be internally consistent in the present study (coefficient omega = 0.75, 95% CI [0.68, 0.81]). Finally, some demographic questions had to be answered. The whole process took 5 min.

### Results

#### Postexperimental Evaluations of Facial Pleasantness and Trustworthiness

To test the validity of our manipulation for facial attractiveness, we first conducted a 2 (attractiveness: attractive, unattractive) ^∗^ 2 (trustworthiness: trustworthy, untrustworthy) between-subjects ANOVA on the postexperimental ratings of pleasantness and trustworthiness. The results revealed a significant main effect of attractiveness [*F*_(1, 3__07__)_ = 72.60, *p <* 0.001, *η*^2^*_*p*_* = 0.19] and trustworthiness [*F*_(1, 3__07__)_ = 25.59, *p <* 0.001, *η*^2^*_*p*_* = 0.08]. Regardless of the level of facial trustworthiness, the attractive faces induced remarkably more pleasantness (*M* = 3.88, *SD* = 1.31) than the unattractive faces (*M* = 2.66, *SD* = 1.37). A significant interaction between attractiveness and trustworthiness [*F*_(1, 3__07__)_ = 17.17, *p <* 0.001, *η*^2^*_*p*_* = 0.05] was also found. The trustworthy face generally evoked greater pleasantness (*M* = 3.64, *SD* = 1.27) than the untrustworthy face (*M* = 2.90, *SD* = 1.56). The rating differences were even greater in the unattractive condition than in the attractive condition (see Table 1).

**TABLE 1:** Mean scores (standard deviations) of postexperimental ratings for facial pleasantness and trustworthiness.

**Independent variables**		**Postexperimental pleasantness**		**Postexperimental trustworthiness**		
**Trustworthiness**	**Attractiveness**	***M***	***SD***	***M***	***SD***	***N***
High	High	3.95	1.19	4.04	1.04	78
	Low	3.33	1.28	3.96	1.32	77
	Total	3.64	1.27	4.00	1.18	155
Low	High	3.82	1.42	3.26	0.99	77
	Low	2.01	1.12	2.82	0.92	79
	Total	2.90	1.56	3.04	0.98	156
Total	High	3.88	1.31	3.65	1.09	155
	Low	2.66	1.37	3.39	1.27	156

Meanwhile, to test the validity of our manipulation for facial trustworthiness, a 2 (attractiveness: attractive, unattractive) ^∗^ 2 (trustworthiness: trustworthy, untrustworthy) between-subjects ANOVA was conducted on the postexperimental ratings of trustworthiness, which revealed significant main effects of trustworthiness [*F*_(1, 307)_ = 61.57, *p <* 0.001, *η*^2^*_*p*_* = 0.17] and attractiveness [*F*_(1, 3__0__7__)_ = 4.43, *p* = 0.036, *η*^2^*_*p*_* = 0.01]. Regardless of the level of facial attractiveness, the trustworthy faces induced significantly more trustworthiness (*M* = 4.00, *SD* = 1.18) than the untrustworthy faces (*M* = 3.04, *SD* = 0.98). No interaction between trustworthiness and attractiveness [*F*_(1, 307)_ = 2.17, *p* = 0.142, *η*^2^*_*p*_* = 0.01] was found.

In general, our manipulation of facial attractiveness and trustworthiness was successful.

#### Conviction and Punishment Ratings

##### *Convictio*n

**FIGURE 1:**
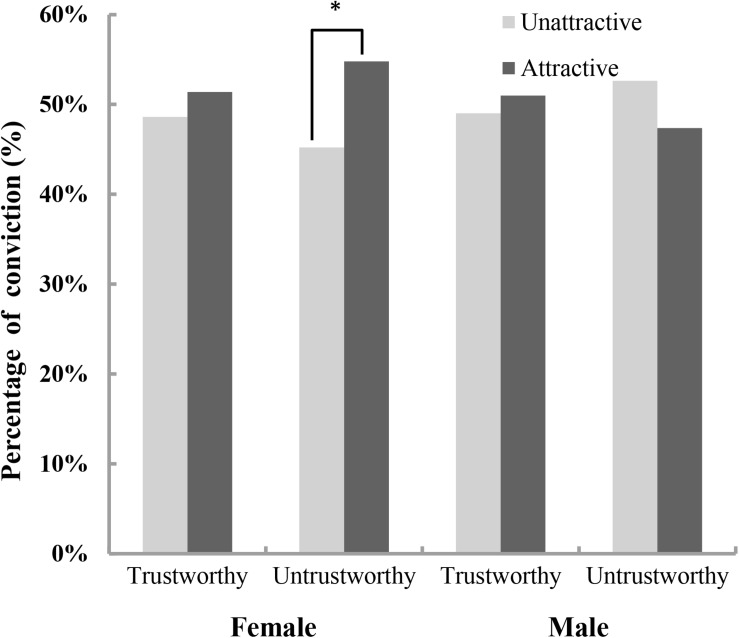
Percentage of convictions in the two trustworthiness conditions for males and females, broken down by attractiveness condition. ^∗^*p* < 0.05.

We calculated the conviction rates in the two trustworthiness conditions for males and females, broken down by attractiveness condition (see Figure 1). There was a significantly greater conviction rate for attractive defendants than for unattractive defendants in the untrustworthy condition among females (*p* = 0.003 for chi-square tests) but not males (*p* = 0.257 for chi-square tests).

We then performed a binary logistic regression, with participants’ gender, facial attractiveness, trustworthiness and their interactions as predictors of conviction. The final model was statistically significant [χ^2^_(__7__, 311)_ = 14.89, *p* = 0.037]. The results also revealed a significant interaction of gender, attractiveness, trustworthiness (Wald = 4.88, *df* = 1, *p* = 0.027, *B* = 3.25, *SE* = 1.47, odds ratio = 25.82, 95% CI [1.44, 462.05]). We further conducted a binary logistic regression for females and males and found that the model was significant for females [χ^2^_(3, 172)_ = 10.75, *p* = 0.013]. In the meantime, there was a significant interaction between attractiveness and trustworthiness among female participants (Wald = 3.92, *df* = 1, *p* = 0.048, *B* = −2.40, *SE* = 1.21, odds ratio = 0.09, 95% CI [0.01, 0.98]). Further analyses showed that the conviction rates were significantly higher for attractive defendants than for unattractive defendants only in untrustworthy conditions (untrustworthy condition: Wald = 5.85, *df* = 1, *p* = 0.016, *B* = 2.59, *SE* = 1.07, odds ratio = 13.33, 95% CI [1.64, 108.70]; trustworthy condition: Wald = 0.11, *df* = 1, *p* = 0.740, *B* = 0.19, *SE* = 0.57, odds ratio = 1.21, 95% CI [0.40, 3.68]). However, the regression model was not significant for males [χ^2^_(3, 139)_ = 1.94, *p* = 0.585], and there was no significant interaction or main effects of facial attractiveness and trustworthiness for male participants (attractiveness^∗^ trustworthiness: Wald = 1.04, *df* = 1, *p* = 0.308, *B* = 0.85, *SE* = 0.83, odds ratio = 2.34, 95% CI [0.46, 12.01]; attractiveness: Wald = 1.26, *df* = 1, *p* = 0.262, *B* = −0.69, *SE* = 0.62, odds ratio = 0.50, 95% CI [0.15, 1.68]; trustworthiness: Wald = 1.54, *df* = 1, *p* = 0.214, *B* = −0.77, *SE* = 0.62, odds ratio = 0.46, 95% CI [0.14, 1.56]).

##### *Punishment magnitud*e

**FIGURE 2:**
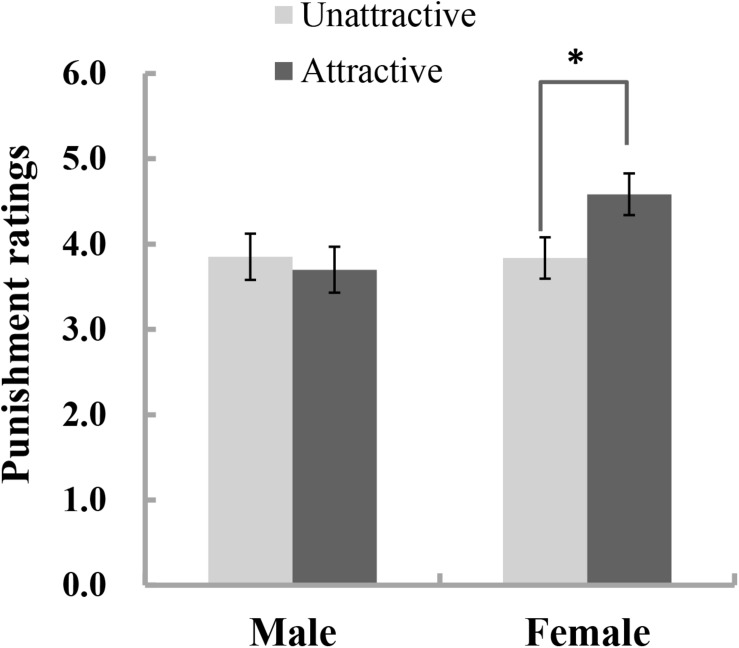
Punishment magnitude ratings that participants chose for defendants with attractive and unattractive faces by collapsing data across the two trustworthiness conditions. Error bars represent ± SE. ^∗^*p* < 0.05.

A 2 (attractiveness: attractive, unattractive) ^∗^ 2 (trustworthiness: trustworthy, untrustworthy) ^∗^ 2 (gender: males, females) three-way ANOVA conducted on the punishment magnitude ratings only revealed a marginally significant interaction effect between gender and attractiveness [*F*_(1, 303)_ = 3.04, *p* = 0.082, *η*^2^_*p*_ = 0.01]. There was no main effect of attractiveness [*F*_(1, 303)_ = 1.33, *p* = 0.250, *η*^2^*_*p*_* < 0.01] or trustworthiness [*F*_(1,303__)_ = 1.43, *p* = 0.232, *η*^2^*_*p*_* = 0.01]. Further tentative analyses for the marginal interaction effect showed that there was a significant effect of facial attractiveness for females [*F*_(1,303__)_ = 4.69, *p* = 0.031, *η*^2^*_*p*_* = 0.02] but not for males [*F*_(1,303__)_ = 0.16, *p* = 0.691, *η*^2^*_*p*_* < 0.01] (see Figure 2). Female participants chose to impose significantly more severe punishments for male defendants with an attractive face (*M* = 4.58, *SD* = 1.90) than for those with an unattractive face (*M* = 3.84, *SD* = 2.37).

#### Correlation Analyses

We did not find any significant correlation between the postexperimental ratings of the two facial dimensions and punishment judgments [pleasantness and punishment judgments (*r* = − 0.05; *p* = 0.361); trustworthiness and punishment judgments (*r* = 0.01; *p* = 0.834)]. The criminal appearance scores tended to have a weak positive correlation with punishment judgments (*r* = 0.12; *p* = 0.036). However, further linear regression analyses did not reveal any significant prediction effect of criminal appearance on punishment magnitude [*b* = 0.23, Δ*R*^2^ = 0.01, *F*_(1, 309)_ = 2.73, *p* > 0.05].

### Discussion

In summary, we found a “beauty-penalty effect” of facial attractiveness mainly for the dichotomous conviction judgments among females, and the effect only occurred when a defendant’s face was untrustworthy. Specifically, female participants were more likely to render a guilty verdict for male defendants with attractive-untrustworthy faces than those with unattractive-untrustworthy faces. This finding was in line with our hypothesis and partly consistent with some of the previous studies (Sigall and Ostrove, 1975; Wuensch et al., 1993; Shechory-Bitton and Zvi, 2014). There might be two possible explanations for the “beauty-penalty effect”. First, in the blind-date swindle, the better-looking male defendants might be considered to have an advantage in a romance and therefore be more dangerous in a romance scam (Sigall and Ostrove, 1975). Second, people have relatively higher expectations for attractive individuals, who are generally assumed to have more positive qualities and more prosocial behaviors (Dion et al., 1972; Langlois et al., 2000). They may receive more condemnation for not living up to people’s expectations of them. In a trust game task, for example, trustees would return significantly less money to the more attractive trusters when the trustees’ higher expectations of their attractive counterparts to send more were not matched (Wilson and Eckel, 2006).

It is noteworthy that the “beauty-penalty effect” among female participants only occurred when a defendant’s face was untrustworthy for the dichotomous conviction judgments. This finding was consistent with our hypothesis. It has been shown that trustworthiness usually contributes to trustworthy trait inferences (Duarte et al., 2012; Ewing et al., 2015), and therefore, a defendant with a trustworthy face may balance out the “beauty-penalty effect” of physical attractiveness on conviction. However, we did not find a significant interaction between attractiveness and trustworthiness for penalty decisions, and the “beauty-penalty effect” was only marginally significant among females. Specifically, females showed a tendency to impose harsher punishments for good-looking defendants regardless of the level of trustworthiness.

Additionally, we did not find any correlation between the postexperimental facial pleasantness ratings and the punishment magnitude ratings. This finding indicates that the punishment magnitude ratings are not explained by a linear increase in facial attractiveness. Instead, people might have dichotomous classification judgments in which good-looking faces deserve relatively harsh punishment, whereas unattractive faces should receive a relatively light penalty. Another possible reason might be related to the fact that we used facial pleasantness instead of facial attractiveness in the correlation analyses. Although facial pleasantness is an emotional indicator of facial attractiveness, it does not fully capture the concept of facial attractiveness. We asked participants to evaluate attractiveness directly in Experiment 2.

## Experiment 2

According to the previous study (Sigall and Ostrove, 1975; Shechory-Bitton and Zvi, 2014), the negative effects of physical attractiveness on judicial decisions should be observed in swindling because the crime was attractiveness-related. However, the results were mixed concerning the effect of facial attractiveness on judicial decisions in swindling in previous studies. Few studies have examined the influence of facial appearances on different types of swindle cases. In Experiment 2, we examined the effect of facial attractiveness and trustworthiness on the judicial decisions of mock jurors in a telecommunication swindle. We adapted the criminal scenario from a real case in which the suspect was accused of swindling a woman out of 10,000 ¥ (approximately $ 1410) by forging online transactions. We believe that it is unlikely for criminals to impress victims with their physical appearance in a telecommunication swindle without them seeing each other. Therefore, the appearance of suspects should not be a factor in their criminal activity. In this case, we hypothesized that better-looking defendants should gain a “leniency effect” in a judicial trial. It is very likely that the “beauty-is-good” stereotype would be activated among judicial decision makers and that facial attractiveness and trustworthiness would combine to contribute to inferences of trustworthy personality traits. The more attractive and trustworthy a person’s face appears, the more favorable the impression he or she may gain from the judicial decision makers. In contrast, defendants with unattractive and untrustworthy faces may be perceived as more criminal in appearance and activate more negative emotion than those with attractive and trustworthy faces. There is evidence suggesting that an untrustworthy face could elicit more negative emotions (Todorov et al., 2008). Moreover, one of our recent studies showed that feelings of disgust toward a criminal better predicted punishment magnitude ratings than did other emotions, such as anger or sympathy (Yang et al., 2019). Therefore, to further reveal the potential mechanism underlying the effect of facial appearances on judicial decisions, we also measured the disgust feelings that participants had toward the defendant in Experiment 2 and conducted an exploratory analysis to examine whether the disgust can play a mediating role in the relationship between facial trustworthiness and the assignment of criminal penalties.

### Materials and Methods

#### Participants

A total of 373 undergraduate and graduate students were recruited in Experiment 2. They were all Asian Chinese, and their ages varied from 17 to 29 years (*M* = 19; *SD* = 1.72). Twenty-one participants did not finish all the questions and were removed from further data analyses, leaving 352 students for the final sample. For the same reason as in Experiment 1, no participant majored in psychology or law. Oral informed consent was provided by each participant. The study followed the ethical guidelines of the Declaration of Helsinki and was approved by the Ethics Committee of Hangzhou Normal University.

#### Materials

The photos and criminal appearance scale used in Experiment 2 were identical to those of Experiment 1. The scale was found to be internally consistent (coefficient omega = 0.79, 95% CI [0.75, 0.83]). For the criminal case, we used a telecommunication fraud case (the detailed criminal case can be seen in the Supplementary Materials), which was also adapted from a real case. The gender of the victim, the amount of loss for the victim, the basic information of the suspect, and the length of the case were matched to the corresponding information in Experiment 1.

#### Design and Procedure

The design in Experiment 2 was the same as that of Experiment 1. There were 89 participants for the attractive and trustworthy face condition (48 females), 89 for the unattractive but trustworthy face condition (47 females), 88 for the attractive but untrustworthy face condition (48 females), and 86 for the unattractive and untrustworthy face condition (45 females).

The procedure of Experiment 2 was the same as that of Experiment 1 except the following: (a) participants needed to give punishment magnitude ratings and sentence the criminal on a scale from 0 to 10 years, with a minimum length of 6 months; (b) participants were asked to directly rate the attractiveness of the face after the legal judgments (postexperimental evaluations); and (c) participants were asked to rate how much aversion they felt toward a defendant’s face once they had completed the criminal appearance scale (postexperimental evaluation).

### Results

#### Postexperimental Evaluations of Facial Attractiveness and Trustworthiness

The 2 (attractiveness) ^∗^ 2 (trustworthiness) between-subjects ANOVA revealed a main effect of attractiveness [*F*_(1, 3__48__)_ = 343.70, *p <* 0.001, *η*^2^*_*p*_* = 0.50]. The main effect of trustworthiness was not significant [*F*_(1, 3__48__)_ = 2.86, *p* = 0.092, *η*^2^*_*p*_* = 0.01]. Regardless of the level of facial trustworthiness, the attractive faces were rated as more attractive (*M* = 3.99, *SD* = 1.21) than the unattractive faces (*M* = 1.83, *SD* = 0.98). However, the interaction between attractiveness and trustworthiness was also significant [*F*_(1, 3__48__)_ = 3.93, *p* = 0.048, *η*^2^*_*p*_* = 0.01]. Attractive and trustworthy faces were rated as less attractive (*M* = 3.98, *SD* = 1.09) than attractive but untrustworthy faces (*M* = 4.01, *SD* = 1.33); unattractive but trustworthy faces were rated as more attractive (*M* = 2.04, *SD* = 1.05) than unattractive and untrustworthy faces (*M* = 1.62, *SD* = 0.84) (see Table 2).

**TABLE 2:** Mean scores (standard deviations) of postexperimental ratings for facial attractiveness and trustworthiness.

**Independent variables**		**Postexperimental attractiveness**		**Postexperimental trustworthiness**		
**Trustworthiness**	**Attractiveness**	***M***	***SD***	***M***	***SD***	***N***
High	High	3.98	1.09	3.79	0.90	89
	Low	2.05	1.05	3.48	0.92	89
	Total	3.01	1.44	3.64	0.92	178
Low	High	4.01	1.34	3.39	0.89	88
	Low	1.62	0.84	2.92	1.01	86
	Total	2.83	1.64	3.16	0.98	174
Total	High	3.99	1.21	3.59	0.91	177
	Low	1.83	0.98	3.21	1.00	175

The 2 (attractiveness) ^∗^ 2 (trustworthiness) between-subjects ANOVA conducted on the postexperimental ratings of trustworthiness revealed significant main effects of trustworthiness [*F*_(1, 3__48__)_ = 23.71, *p <* 0.001, *η*^2^*_*p*_* = 0.06] and attractiveness [*F*_(1, 3__48__)_ = 15.15, *p <* 0.001, *η*^2^*_*p*_* = 0.04]. Regardless of the level of attractiveness, the trustworthy faces were rated as more trustworthy (*M* = 3.63, *SD* = 0.92) than the untrustworthy faces (*M* = 3.16, *SD* = 0.98). There was no significant interaction between the two facial dimensions [*F*_(1, 3__48__)_ = 0.69, *p* = 0.407, *η*^2^*_*p*_* < 0.01].

These postexperimental rating results indicated that the manipulation of the two facial dimensions was successful in general.

#### Conviction and Punishment Ratings

##### *Convictio*n

As in Experiment 1, we calculated the conviction rates in the two trustworthiness conditions for males and females, broken down by attractiveness condition. Regardless of the trustworthiness, there were no significant differences in conviction rates between attractive and unattractive faces for male or female participants (*p*s > 0.1 for all chi-square tests).

A binary logistic regression was then performed with participants’ gender, facial attractiveness, trustworthiness and their interactions as predictors of conviction. The final model was not significant [χ^2^_(7, 352__)_ = 5.36, *p* = 0.616], and there was no main effect of gender, attractiveness or trustworthiness (gender: Wald = 0.25, *df* = 1, *p* = 0.620, *B* = −0.25, *SE* = 0.50, odds ratio = 0.78, 95% CI [0.29, 2.09]; attractiveness: Wald = 1.41, *df* = 1, *p* = 0.235, *B* = −0.56, *SE* = 0.47, odds ratio = 0.57, 95% CI [0.23, 1.44]; trustworthiness: Wald = 2.12, *df* = 1, *p* = 0.145, *B* = −0.69, *SE* = 0.47, odds ratio = 0.50, 95% CI [0.20, 1.27]).

##### *Punishment magnitud*e

A similar ANOVA was conducted on the punishment magnitude ratings. Gender [*F*_(1, 3__44__)_ = 0.06, *p* = 0.801, *η*^2^*_*p*_* < 0.01 (*M*_m_ = 3.31, *SD* = 2.51; *M*_f_ = 3.38, *SD* = 2.53)], attractiveness [*F*_(1, 3__44__)_ = 0.34, *p* = 0.561, *η*^2^*_*p*_* < 0.01 (*M*_H =_ 3.42, *SD* = 2.53; *M*_L_ = 3.26, *SD* = 2.52)] and trustworthiness [*F*_(1, 3__44__)_ = 0.33, *p* = 0.564, *η*^2^*_*p*_* < 0.01 (*M*_H_ = 3.26, *SD* = 2.60; *M*_L_ = 3.42, *SD* = 2.44)] had no main effect on punishment magnitude. No other significant effect was found (*p*s > 0.1).

##### *Sentencing decision*s

Similarly, we did not find any main effect of gender [*F*_(1, 3__44__)_ = 0.01, *p* = 0.777, *η*^2^*_*p*_* < 0.01 (*M*_m_ = 0.99, *SD* = 1.48; *M*_f_ = 0.95, *SD* = 1.53)], attractiveness [*F*_(1, 3__44__)_ < 0.01, *p* = 0.963, *η*^2^*_*p*_* < 0.01 (*M*_H_ = 0.96, *SD* = 1.41; *M*_L_ = 0.98, *SD* = 1.60)] or trustworthiness [*F*_(1, 3__44__)_ = 0.32, *p* = 0.570, *η*^2^*_*p*_* < 0.01 (*M*_H_ = 0.92, *SD* = 1.41; *M*_L_ = 1.01, *SD* = 1.59)]. There was also no interaction among these variables (*p* = 0.790).

#### Correlation Analyses

We observed several significant correlations between facial variables and legal judgments (see Table 3). A binary logistic regression analysis further revealed that trustworthiness ratings significantly predicted the dichotomous verdict (*B* = −0.52, *SE* = 0.13, Wald = 16.04, *p*< 0.001, odds ratio = 0.59, 95% CI [0.46, 0.77]). Meanwhile, trustworthiness was also a significant predictor for punishment magnitude ratings [*b* = −0.58, Δ*R*^2^ = 0.05, *F*_(1, 35__0__)_ = 18.76, *p*< 0.001] and sentencing decisions [*b* = −0.34, Δ*R*^2^ = 0.05, *F*_(1, 35__0__)_ = 17.93, *p*< 0.001] in linear regression analyses.

**TABLE 3:** Zero-order correlations among the variables of interest (*N* = 352).

	**1**	**2**	**3**	**4**	**5**	**6**	**7**
1. Attractiveness	1.000						
2. Trustworthiness	0.31^∗∗∗^	1.000					
3. Criminal appearance	–0.37^∗∗∗^	–0.61^∗∗∗^	1.000				
4. Disgust	–0.37^∗∗∗^	–0.34^∗∗∗^	0.61^∗∗∗^	1.000			
5. Verdict	–0.03	–0.21^∗∗∗^	0.10	0.03	1.000		
6. Punishment ratings	–0.03	–0.20^∗∗∗^	0.15^∗∗^	0.14^∗∗^	0.82^∗∗∗^	1.000	
7. Sentences	–0.07	–0.19^∗∗∗^	0.14^∗∗^	0.18^∗∗^	0.69^∗∗∗^	0.74^∗∗∗^	1.000

**^∗∗^p < 0.01 and ^∗∗∗^p < 0.001.**

#### Mediation Analyses

We conducted mediation analyses through the PROCESS macro for SPSS (Hayes, 2013) to test whether the effect of facial trustworthiness on sentencing decisions was mediated via emotion responses.

**FIGURE 3:**
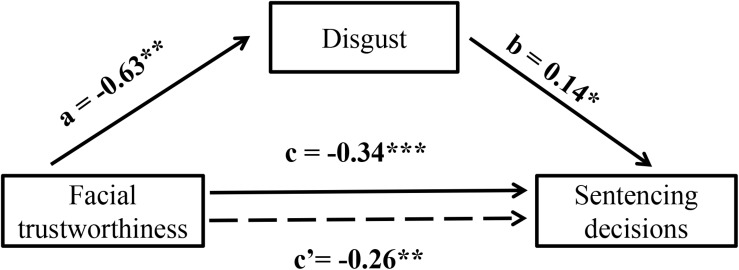
The mediation analyses of the disgust emotion between facial trustworthiness ratings and sentencing decisions. ^∗^*p* < 0.05, ^∗∗^*p* < 0.01, and ^∗∗∗^*p* < 0.001.

As the results showed, both the direct effect of facial trustworthiness on sentencing decisions and the indirect effect of facial trustworthiness through emotion responses were significant, indicating that disgust might partially mediate the relationship between facial trustworthiness and sentencing decisions. The point estimate of indirect of facial trustworthiness on sentencing decisions was −0.08 (95% confidence interval: −0.71 to −0.01), and the point estimate of the direct effect of facial trustworthiness on sentencing decisions was −0.26 (95% confidence interval: −0.43 to −0.08) when disgust was controlled (see Figure 3 for the mediation model).

### Discussion

Unlike the blind-date swindle in Experiment 1, we did not find any effect of attractiveness on the judicial decisions in the telecommunication swindle. However, we did find a leniency effect of trustworthiness. Specifically, the more trustworthy a face looked, the less severe the criminal penalties a defendant received. Previously, it was demonstrated that facial trustworthiness biased judicial decisions only in major crimes (Porter et al., 2010). The circumstances of the crimes in the present study should be considered minor, and surprisingly, there was still a significant linear relationship between facial trustworthiness and judicial decisions. This finding further suggests that the nature of the offenses has a bearing on the relationship between facial trustworthiness and judicial decision making. Furthermore, the results of an exploratory analysis revealed that disgust partially mediated the effect of facial trustworthiness on judicial decisions.

## General Discussion

This study examined the influences of facial appearance features on judicial decisions in two different swindles by simultaneously manipulating facial attractiveness and trustworthiness. This research mainly extends the findings of previous research in the following two aspects.

First, our findings support the “beauty-penalty effect” of facial attractiveness, which was reported by some previous studies (Sigall and Ostrove, 1975; Wuensch et al., 1993; Shechory-Bitton and Zvi, 2014). Furthermore, we also revealed an interactive effect between facial attractiveness and trustworthiness during judicial decisions in the blind-date swindle. The conviction rate was significantly higher for attractive defendants than for unattractive defendants only in the untrustworthy condition. This result may suggest that those who failed to find any effects of facial attractiveness on the judicial decision could be because they focused on manipulating facial attractiveness without controlling for facial trustworthiness (Smith and Hed, 1979; Mazzella and Feingold, 1994). Despite the fact that this interactive effect was merely observed in the conviction judgments, our findings provided some evidence that facial traits might interactively influence judicial decisions in certain cases and that carefully matching other facial features is necessary when focusing on one facial feature during judicial decisions.

Second, we demonstrated that the effects of facial appearance on judicial decisions could vary significantly among different criminal scenarios for the same type of swindles. It has been well recognized since the early studies that the effect of facial appearance on judicial decisions might differ as the nature of the crimes changes (Sigall and Ostrove, 1975). However, previous studies have mostly focused on different types of crimes (for example, swindle or burglary) when discussing crime features. Few have investigated how the effect of facial appearance on criminal punishments might differ in the same crimes of swindling that were generally believed to be attractiveness-related. Our results showed that the same male face for a defendant biased criminal punishment outcomes differently in romance-related and telecommunication-related swindles. To be more specific, in the blind-date swindle, there was a “beauty-penalty effect” for the conviction judgments. However, in the telecommunication swindle, facial trustworthiness rather than facial attractiveness significantly predicted criminal penalties. These results suggest that the details of the criminal circumstances in addition to the general crime types mattered for the distinct effects of facial features on legal decisions.

Moreover, our findings suggested different underlying mechanisms for the impact of facial attractiveness and trustworthiness on judicial decisions. The “beauty-penalty effect” for the attractive faces in the blind-date swindle was possibly due to the dashed expectations participants might have for the better-looking defendants. In that case, it should be reasonable to expect a similar “beauty penalty” for the attractive faces in the telecommunication swindle, which used the same photos for the defendants. Since no significant effect of facial attractiveness was observed in Experiment 2, we have reason to believe that the attractive defendants were punished more in the blind-date swindle than the unattractive defendants were because the romance-related criminal circumstances easily activated people’s stereotypes that lead them to believe that the more attractive a defendant is, the more dangerous he might be in a romance-related swindle. However, the “leniency effect” for facial trustworthiness might be more likely due to trustworthy trait inferences and the role of emotions. The trustworthiness of a face accounted for a large proportion of criminal appearances (Flowe, 2012). In fact, facial trustworthiness is an essential component of criminal appearance that is proportional to the magnitude of punishment and the length of a prison term in the telecommunication swindle (see the Supplementary Results).

Additionally, the exploratory analysis in Experiment 2 revealed that disgust can partially mediate the relationship between trustworthiness and judicial decisions. As a typical threat-related emotion, disgust plays the evolutionary role of helping us avoid harmful substances (Rozin et al., 1999). Some studies have revealed the role of disgust in the legal domain. For example, the conviction rate was significantly higher when gruesome and disgusting photographs were included (Bright and Goodman-Delahunty, 2006). Participants who were exposed to strong disgust were motivated to punish a defendant more severely (Capestany and Harris, 2014). Our results indicated that untrustworthy faces predicted harsher penalties. This may be due to in part the fact that those faces evoke aversive emotions toward a criminal defendant. Although the mediation analysis of disgust in the present study was just exploratory, it provides some explanations for the “leniency effect” of facial trustworthiness in the telecommunication swindle.

Above all, the results from the two experiments suggested that facial appearance does make a difference in mock juries’ decision making. In particular, a face congruent with an alleged crime could put a defendant at a disadvantage during the judicial process (Shoemaker et al., 1973; Bull and Green, 1980; Macrae and Shepherd, 1989; Yarmey, 1993; Dumas and Testé, 2007).

However, it is important to note that only female participants were affected by facial attractiveness during their decision making in the blind-date swindle. We did not find any significant differences in postexperimental facial pleasantness ratings between males and females (results are available in Supplementary Materials); thus, we can exclude the possibility that the gender effect in judicial decision making was due to gender-based perceived differences in facial attractiveness. Instead, we assume that the gender differences might be explained by the defensive attribution theory (Shaver, 1970) to some extent. According to the theory, people tend to reduce their blame on those with whom they identify to defend themselves against similar negative life situations that might happen to them in the future. The criminal scenarios in the current study always involve a male defendant and a female victim. It is very likely that female participants might empathize with the female victims more than male participants would and clearly see their vulnerability to the crimes involving a male swindler. This attribution bias of blame among female participants may facilitate the activation of the stereotypes that the better-looking male defendant could easily take advantage of his physical appearance to deceive female victims. As we failed to observe a similar pattern in the telecommunication swindle case, these findings suggest that the gender effect for the impact of facial attractiveness on judicial decisions also hinges on the nature of the crimes.

Some limitations of this study need to be addressed. First, although our manipulations of facial attractiveness and trustworthiness were successful in general, we found it very difficult to match attractiveness or trustworthiness in different conditions due to an intertwined relationship between the two facial traits. For instance, an unattractive face was always rated as more attractive in the trustworthy condition than in the untrustworthy condition. In addition, despite our efforts to select an attractive or trustworthy face with extreme values, the maximum rating values for a physically attractive or trustworthy face were moderately high. This may reduce the differences in the facial effect on judicial decisions between an attractive and unattractive face or a trustworthy and untrustworthy face. Considering that the two facial features might not be separable on the objective level, we may try to train participants on how to perceive the photos. For example, participants could be trained to categorize photos into trustworthy and untrustworthy faces based on personal experiences as there has been solid evidence suggesting that individuals’ conceptual beliefs and past experiences influence facial trustworthiness judgments through an associative learning mechanism (Falvello et al., 2015; FeldmanHall et al., 2018; Stolier et al., 2018). Second, we merely observed a significant interactive effect of facial attractiveness during conviction judgments in Experiment 1. Other studies have demonstrated a facial effect during sentencing decisions (Stewart, 1980, 1985; Umukoro and Egwuonu, 2014), and previous evidence has also suggested that not only the conviction but also the sentencing could be affected by facial attractiveness (Efran, 1974). Thus far, we are not able to draw any clear conclusions regarding the question of whether facial attractiveness would take effect in a certain judicial stage or during the whole judicial process. Third, we only used male defendants in the present study. Further studies are needed to include female defendants as well and see how participants would be responsive to female defendants’ facial features. Finally, we collected our data in a sample of non-law college students. It remains unknown whether the findings we observed in the current study would be tempered by professional knowledge and expertise. Additionally, in real trials, the juries normally reach a final verdict based on group decisions. Therefore, we should be cautious in our application of the current findings to courtrooms.

Overall, our results showed that the impact of physical appearances on penalty decision-making in swindling cases differs as the detailed criminal circumstances change. An attractive but untrustworthy face in the blind-date swindle might put a male defendant at a disadvantage when the judicial decision makers are females. However, a trustworthy male face in the telecommunication swindle might lead to a more lenient penalty. It should be noted that despite the consistent findings of the physical appearance stereotypes in social decision making, no relationship between the facial features and the actual personality traits has been found (Rule et al., 2013). An attractive or trustworthy face is not necessarily related to a specific personality trait. Therefore, it is highly risky to make real-life decisions based on subjective impressions of one’s physical appearances (Porter et al., 2008).

## Data Availability Statement

All datasets generated for this study are included in the manuscript/Supplementary Files.

## Ethics Statement

This study was carried out in accordance with the recommendations of the Ethics Committee of Hangzhou Normal University, Institutional Review Board of Hangzhou Normal University; with oral informed consent from all subjects. All subjects gave oral informed consent in accordance with the Declaration of Helsinki. The protocol was approved by Hangzhou Normal University.

## Author Contributions

QY developed this study. QZ and YW prepared the experimental materials. BZ, RH, and SL collected the data. BZ performed the data analyses and drafted the initial manuscript. QY and DS made critical revision and polished the manuscript. All the authors have read the final version of the manuscript and approved its publication.

## Conflict of Interest

The authors declare that the research was conducted in the absence of any commercial or financial relationships that could be construed as a potential conflict of interest.

## Footnotes

1 https://www.wjx.cn/app/survey.aspx
