# Supplementary material for: Effects of Male Defendants’ Attractiveness and Trustworthiness on Simulated Judicial Decisions in Two Different Swindles
Source: Front Psychol. 2019 Sep 26;10:2160. doi: 10.3389/fpsyg.2019.02160 (PMC6775219; doi:10.3389/fpsyg.2019.02160)
Supplement: Supplementary file 1 [file Table_1.docx]

**Supplementary Materials**

1. **Supplementary Results**

**1.1 Confidence in Conviction in Experiment 1**

**1.1.1 Un-transformed Confidence Results**

A three-way ANOVA with all the variables as the between-subject factors was carried out on the original confidence scores. We found an interaction effect of gender and facial attractiveness [*F*_(1, 303)_ = 5.63, *p* = 0.018, *η^2^_p_* = 0.02]. There was no main effect of attractiveness [*F*_(1, 303)_ = 2.15, *p* = 0.144, *η^2^_p_* = 0.01] and trustworthiness [*F*_(1, 303)_ = 0.05, *p* = 0.818, *η^2^_p_* < 0.01]. Further simple effect analyses for the interaction showed that there was a significant effect of facial attractiveness for males [*F*_(1, 303)_ = 6.66, *p* = 0.010, *η^2^_p_* = 0.02] but not for females [*F*_(1, 303)_ = 0.46, *p* = 0.498, *η^2^_p_* < 0.01]. Males were significantly more confident in their judgments on unattractive defendants (*M* = 5.77, *SD* = 1.09) than attractive defendants (*M* = 5.20, *SD* = 1.50) whether they gave a guilty verdict or not. There was no significant difference of confidence between attractive and unattractive faces among female participants (attractive face: *M* = 5.22, *SD* = 1.22; unattractive face: *M* = 5.09, *SD* = 1.33).

**1.1.2** **Transformed Confidence Results**

As the original conviction confidence did not distinguish between guilty and not guilty decisions. For example, if a participant indicated 5 in the ratings of conviction confidence, this could mean that the confidence that he believes the defendant guilty is 5, or that the confidence that the defendant is not guilty is 5. We therefore adopted a more sensitive measure ([Kassin and Sommers, 1997](#_ENREF_15)) to examine participants’ confidence in conviction, we first re-encoded the dichotomous variable (guilty = 1, not guilty = −1) and then multiplied it by participants’ initial conviction confidence (1: not confident at all; 7: extremely confident), which yielded a new continuous variable (from −7 to 7). A three-way ANOVA was carried out again on the converted confidence scores. The results yielded an interaction effect of gender and facial attractiveness [*F*_(1, 303)_ = 3.95, *p =* 0.048, *η^2^_p_* = 0.01]. The main effect of attractiveness and trustworthiness were not significant [*F*_(1, 303)_ = 0.38, *p =* 0.537, *η^2^_p_* < 0.01; *F*_(1, 303)_ = 0.80, *p =* 0.373, *η^2^_p_* < 0.01]. Further simple effect analyses for interaction effect showed that there was a significant effect of facial attractiveness for females [*F*_(1, 303)_ = 4.76, *p =* 0.031, *η^2^_p_* = 0.03] but not for males [*F*_(1, 303)_ = 0.40, *p =* 0.528, *η^2^_p_* < 0.01] (see Supplementary Figure 1). Females were significantly more confident in convicting a male defendant with an attractive face (*M* = 4.45, *SD* = 3.05) than those with an unattractive face (*M* = 3.23, *SD* = 4.18).

**1.2 Confidence in Conviction in Experiment 2**

**1.2.1 Un-transformed Confidence Results**

The 2*2*2 ANOVA showed an interaction effect of gender and trustworthiness [*F*_(1, 344)_ = 4.84, *p =* 0.028, *η^2^_p_* = 0.01]. Further simple effect analyses for the interaction showed that there was a significant main effect of facial trustworthiness for males [*F*_(1, 344)_ = 6.73, *p =* 0.010, *η^2^_p_* = 0.04]. Specifically, males have significant more confidence in their judgments on untrustworthy defendants than trustworthy defendants (untrustworthy face: *M* = 5.24, *SD* = 1.36; trustworthy face: *M* = 4.71, *SD* = 1.23).

**1.2.2 Transformed Confidence Results**

The 2*2*2 ANOVA was conducted on the transformed confidence again. However, the results did not show any main effect of attractiveness [*F*_(1, 344)_ = 0.02, *p =* 0.894, *η^2^_p_* < 0.01] and trustworthiness on confidence [*F*_(1, 344)_ = 2.46, *p =* 0.117, *η^2^_p_* = 0.01]. There was no interaction effect between attractiveness and trustworthiness [*F*_(1, 344)_ = 3.20, *p* < 0.050, *η^2^_p_* = 0.01].

**1.2.3 Further Analyses of Criminal Appearance**

As a reverse coded item of criminal appearance, trustworthiness was negatively correlated with criminal appearance (r = − 0.61, *p* < 0.001). Contrary to trustworthiness, criminal appearance positively predicted punishment magnitude ratings [*b* = 0.49, Δ*R*^2^ = 0.03, *F*_(1, 350)_ = 10.44, *p* = 0.001] and sentencing decisions [*b* = 0.30, Δ*R*^2^ = 0.03, *F*_(1, 350)_ = 10.10, *p* = 0.001]. We further divided participants into two groups (high-score group and low-score group) based on the criminal appearance rating scores of the photos using the median-split method (*M_d_* = 4.00). Finally, an independent *t*-test showed that the difference of punishment magnitudes [*t* = −2.91, *p* = 0.004, and Cohen’*d* = 0.31 (*M_L_* = 2.97, *SD* = 2.44; *M*_H_ = 3.74, *SD* = 2.55)] and sentencing decisions between the two groups were significant [*t* = −3.30, *p* = 0.001 and Cohen’*d* = 0. 35 (*M*_L_ = 0.72, *SD* = 1.16; *M*_H_ = 1.24, *SD* = 1.76)]. Apparently, defendants with a criminal appearance were at a disadvantage (see Supplementary Figure 2).

**1.3 Gender Differences in the Postexperimental Evaluations**

An independent t-test was conducted to examine whether there is any gender differences in the postexperimental evaluations of facial attractiveness and trustworthiness (see Supplementary Table 1). We did not find any significant differences in the postexperimental facial attractiveness ratings between males and females either in the blind-date swindle (pleasantness: *t* = 1.51, *df* = 309, *p* = 0.131; trustworthiness: *t* < 0.01, *df* = 309, *p* = 0.833) or in the telecommunication swindle (attractiveness: *t* = 0.21, *df* = 350, *p* = 0.997; trustworthiness: *t* = 1.62, *df* = 309, *p* = 0.106).

- 1. **Collapsing Data across the Two Studies**

We tentatively collapsed the data from the two scenarios and performed a binary logistic regression for conviction judgments in an attempt to reveal whether there was an interactive effect between the facial features and the criminal scenarioss among female samples. We found that the model was significant [χ^2^ _(7, 360)_ = 24.84, *p* = 0.001]. The interaction effect of the criminal scenarios, facial attractiveness and trustworthiness was significant: Wald = 5.38, *df* = 1, *p* = 0.020, *B* = -3.18, *SE* =1.37, odds ratio = 0.04, 95% CI [0.003, 0.61].

Further binary logistic regression analyses with participants’ facial attractiveness, trustworthiness, and their interactions as predictors of conviction were conducted for each of the two criminal scenarios. The results could be found in the main manuscript.

1. **Supplementary Figures and Tables**

**2.1 Supplementary Figures**


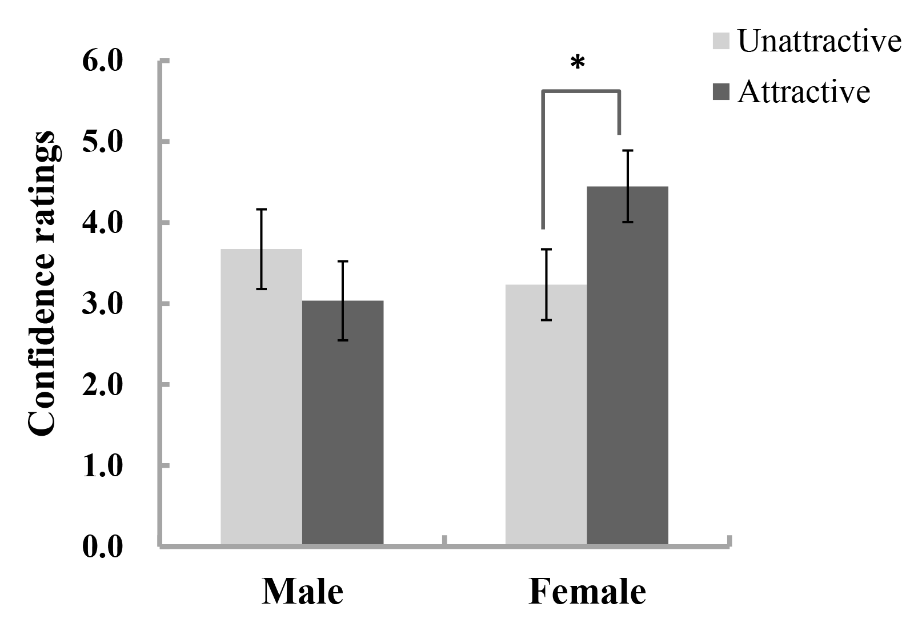


**Supplementary Figure 1.** Transformed confidence of participants in attractive and unattractive faces among males and females by collapsing data across the trustworthiness condition. Error bars represent ± SE. **p* < 0.05; ***p* < 0.01.


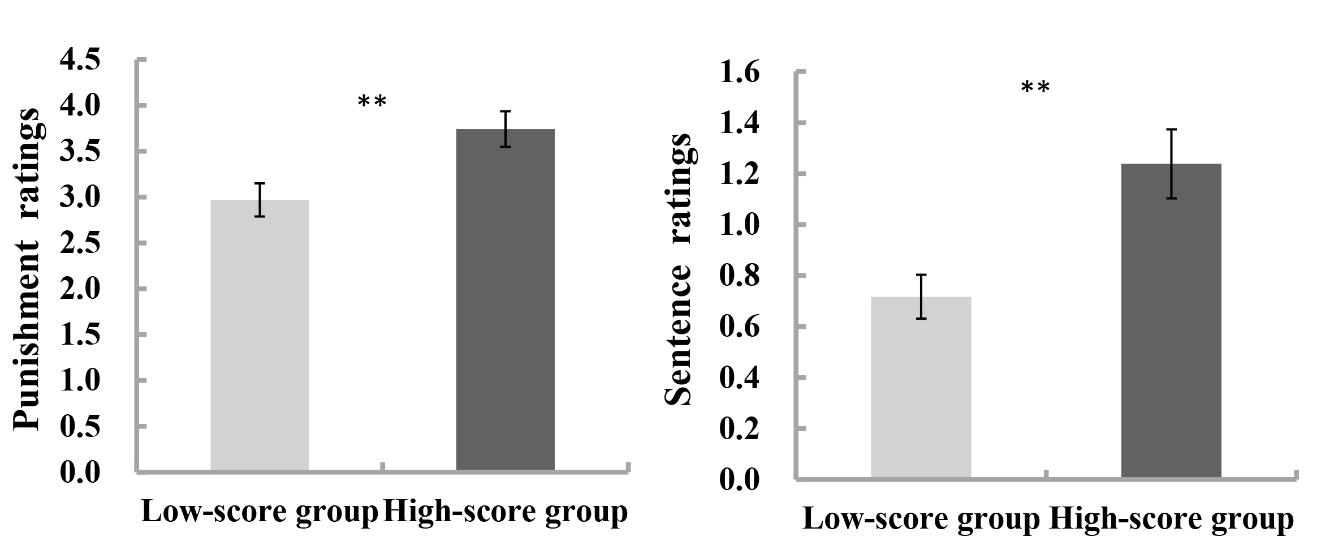


**Supplementary Figure 2.** The punishment and sentencing decision ratings of low-score and high-score groups of criminal appearance. Error bars represent ± SE. **p* < 0.05; ***p* < 0.01.

**2.2 Supplementary Tables**

**Supplementary Table 1. Mean scores (standard deviations) of postexperimental ratings for facial attractiveness and trustworthiness between male and female participants.**

| **Criminal case** | **Facial feature** | **Gender** | ***M*** | ***SD*** | ***N*** |
| --- | --- | --- | --- | --- | --- |
| Blind-date swindle | pleasantness | male | 3.41 | 1.44 | 139 |
|  |  | female | 3.16 | 1.48 | 172 |
|  | trustworthiness | male | 3.52 | 1.12 | 139 |
|  |  | female | 3.52 | 1.24 | 172 |
| Telecommunication swindle | attractiveness | male | 2.94 | 1.52 | 164 |
|  |  | female | 2.90 | 1.57 | 188 |
|  | trustworthiness | male | 3.49 | 1.01 | 164 |
|  |  | female | 3.32 | 0.94 | 188 |

**3 Criminal Cases**

**3.1 Blind-date swindle**

In October 2009, a woman called the Police and stated the case: she got acquainted with a man (Mr. A) online, and they managed to develop a close relationship after dating several times. Two weeks later, the man borrowed 10,000 ¥ (approximately $ 1410) from the woman on the excuses of company bankruptcy and his father’s suffering from a car accident. The woman asked Mr. A to make a receipt after lending money to him, but Mr. A refused for various reasons. The woman also mentioned that almost all the information provided by Mr. A was fabricated. She once tried to call Mr. A and went to his residence, but she could not get in touch with him anymore. The police found that Mr. A had no formal occupation by visiting his neighbors. According to the woman’s description and previous investigation, the police arrested Mr. A as a suspect.

The information provided by the police about Mr. A: male, 29 years old (Han), a college diploma.

**3.2 Telecommunication swindle**

In March 2014, a woman called the Police and described that she received a call from a man who claimed to be an online shop customer service and said that there was a quality problem for the shoes purchased by the woman. So, the woman needed to offer her account to get a full refund. The woman did buy sneakers for her husband two weeks ago. When she asked why the refund could not be returned through the official website, the man explained that the online transaction had been closed. Since the man provided enough detailed information, the woman did not suspect him too much. Then the woman clicked the link sent by the man and found that her 10,000 ¥ (approximately $ 1410) was all transferred. After having investigated the account verification and fund transaction, the police found that the account owner Mr. A, being unemployed, had frequent transaction records, including a record of 10,000 ¥. According to the woman's description and preliminary investigation, the police arrested Mr. A as a suspect. However, Mr. A said that he had lent his account to a friend long ago, and he did not know what his friend had done with the account.

The information provided by the police about Mr. A: male, 29 years old (Han), a college diploma.

**References**

Kassin, S.M., and Sommers, S.R. (1997). Inadmissible testimony, instructions to disregard, and the jury: Substantive versus procedural considerations. *Personality and Social Psychology Bulletin* 23**,** 1046-1054. doi: 10.1177/01461672972310005.
